# Clinical and molecular characterization of non-*PIK3CA*-related vascular malformations with overgrowth in a pediatric cohort

**DOI:** 10.1007/s12519-026-01098-3

**Published:** 2026-09-30

**Authors:** Giuseppe Reynolds, Maria Luca, Paola Coppo, Roberta La Selva, Chiara Leoni, Iacopo Bellani, Simona Ragusa, Stefania Massuras, Sonia Perrelli, Stefania Martino, Paola Sabrina Buonuomo, Federico Rondot, Alessandro Mauro Spinelli, Carla Morando, Marilidia Piglionica, Nicoletta Resta, Alessandro Mussa

**Affiliations:** 1 Department of Public Health and Pediatrics, University of Turin, Turin, Italy; 2 Postgraduate School of Pediatrics, Department of Public Health and Pediatrics, University of Turin, Turin, Italy; 3 Ph.D. Program in Biomedical Sciences and Oncology, Department of Public Health and Pediatrics, University of Turin, Turin, Italy; 4 Pediatric Clinical Genetics, Regina Margherita Children’s Hospital, Turin, Italy; 5 Pediatric Dermatology, Regina Margherita Children’s Hospital, Città della Salute e della Scienza di Torino, Turin, Italy; 6 Department of Woman and Child Health and Public Health, Center for Rare Diseases and Birth Defects, Fondazione Policlinico Universitario “A. Gemelli” IRCCS, Rome, Italy; 7 School of Medicine, University of Turin, Turin, Italy; 8 Medical Genetics Unit, Department of Precision and Regenerative Medicine and Ionian Area (DiMePRe-J), University of Bari “Aldo Moro”, Bari, Italy; 9 Rare Diseases and Medical Genetics Unit, Bambino Gesù Children’s Hospital IRCCS, Rome, Italy; 10 Regional Coordination Center for Rare Diseases, Udine, Italy; 11 Department of Pediatrics, San Bortolo Hospital, Vicenza, Italy

**Keywords:** Children, Vascular malformations, PIK3CA/mTOR pathway, Overgrowth

## Abstract

**Introduction:**

Vascular malformations (VMs) associated with overgrowth represent a heterogeneous group of congenital disorders driven by somatic or germline variants in genes regulating vascular morphogenesis and cellular proliferation. While *PIK3CA*-related overgrowth spectrum (PROS) is relatively well characterized, non-*PIK3CA* VMs with overgrowth remain poorly defined, with limited genotype-phenotype correlations and no standardized therapeutic strategies. This study aimed to characterize the clinical and molecular features of this underrecognized group in a pediatric cohort.

**Methods:**

We conducted a multicentric retrospective study across five Italian tertiary centers. Patients were included if they presented with a vascular malformation, clinically appreciable regional overgrowth beyond the vascular lesion itself, and a confirmed pathogenic or likely pathogenic variant in genes of the PI3K/AKT/mTOR or related signalling pathways, excluding *PIK3CA*. Genetic testing was performed via next-generation sequencing (NGS) using a 17-gene targeted panel (read depth >2000 ×) on peripheral blood, buccal swab, or affected-tissue biopsies. Clinical data were systematically collected across six predefined domains including phenotype, imaging, hemostatic parameters, and therapeutic management.

**Results:**

A total of 41 patients were enrolled, including 20 males and 21 females. The mean age at diagnosis was 12.1 ± 1.4 years. Variants were identified in *TEK* (24.4%), *GNAQ* (21.9%), *GNA11* (21.9%), *PTEN* (14.6%), *RASA1* (12.2%), *AKT3* (2.4%) and *EPHB4* (2.4%). Somatic variants accounted for 65.9% of cases. Phenotypic features included skeletal anomalies (46.3%), soft tissue overgrowth (60.9%), macrocrania (19.5%), and segmental undergrowth in a subset. A syndromic diagnosis was established in 63.4% of patients; however, marked phenotypic overlap across genes was observed. Functional limitations affected 43.9% of patients. Alpelisib yielded clinical improvement in selected TEK-variant patients, whereas sirolimus showed limited efficacy.

**Conclusions:**

Non-*PIK3CA* VMs with overgrowth constitute a clinically and molecularly heterogeneous spectrum with substantial phenotypic overlap across genotypes, rendering phenotype-based classification unreliable. Somatic mosaicism, variable allele frequency, and tissue-specific expression underlie this heterogeneity. Our findings advocate for broad NGS panels with tissue-based analysis, systematic parental testing, and molecularly driven classification. The observed response to alpelisib in *TEK*-related disease supports the concept of convergent downstream pathway activation as a rationale for targeted therapies beyond PROS.

**Graphical abstract:**

**Figure:**
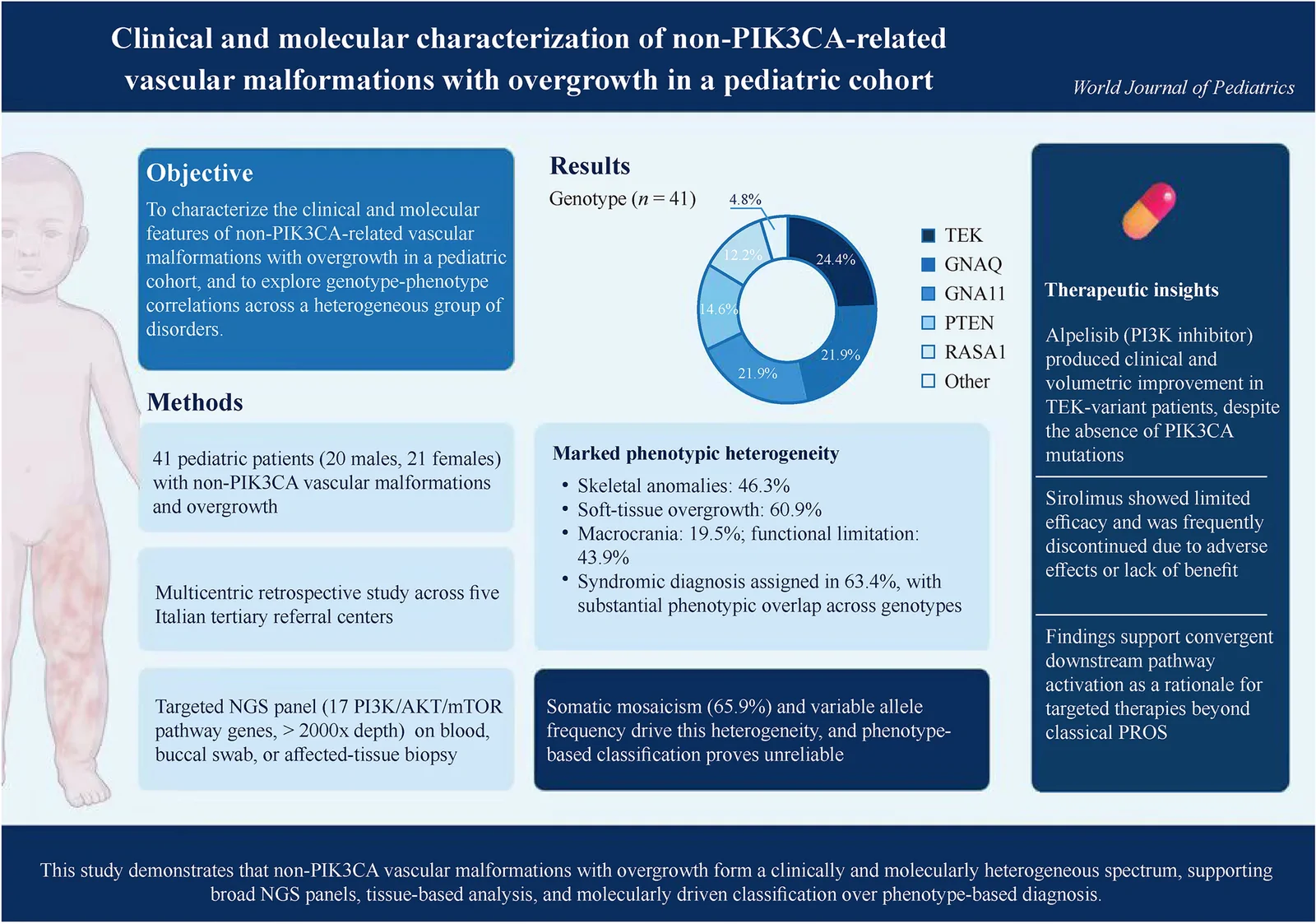

## Introduction

Vascular malformations (VMs) are congenital anomalies of the vascular system arising from errors in vascular morphogenesis [1]. According to the classification proposed by Mulliken and Glowacki in 1982, VMs are structural lesions present at birth—although they may become clinically evident later—that typically grow proportionally with the individual. Histologically, they are characterized by a normal endothelial cell architecture, reflecting a normal endothelial turnover, despite the potential for significant and progressive clinical manifestations [2].

VMs are classified based on flow characteristics and vessel type involved, including capillary, venous, lymphatic, arteriovenous, or combined, and exhibit a wide range of clinical presentations, from asymptomatic cutaneous lesions to life-threatening complications, such as bleeding, thrombosis, and cardiac overload [3, 4]. This disease can occur isolated or as part of complex phenotypes involving tissue overgrowth [5], a disproportionate enlargement of body regions, either focal or segmental, resulting from post-zygotic (somatic) variants that affect the regulation of cellular proliferation, growth, and differentiation. Overgrowth can involve various tissues (adipose, muscular, skeletal, and cutaneous) and usually has a mosaic distribution pattern. The clinical impact of overgrowth can range from mild asymmetry barely appraisable at clinical evaluation to severe deformities and functional impairment, significantly contributing to patient morbidity and requiring multidisciplinary management [5].

Many VMs, associated or not with overgrowth, are caused by somatic variants in genes within the PI3K-AKT-mTOR and RAS-MAPK signalling pathways. Mutations in *PIK3CA* are among the most commonly identified and studied, defining a group of disorders collectively known as the *PIK3CA*-related overgrowth spectrum (PROS) [6]. Many VMs have a molecular basis distinct from PROS and do not meet its clinical criteria, representing a heterogeneous and still poorly characterized group. Genotype-phenotype correlations are limited by disease rarity, technical challenges in detecting low-level mosaicism, and biological variability. The main genetic drivers involve genes regulating angiogenesis and vascular proliferation, such as *GNAQ*, *GNAS*, *GNA11*, *RASA1*, and *TEK*. Although a proportion of cases remain molecularly unresolved despite extensive testing [7]. Most variants are somatic gain-of-function events, paralleling the molecular mechanisms observed in *PIK3CA*-related disorders. In clinical practice, these conditions often present with phenotypes that substantially overlap with entities within the PROS spectrum, and in a subset of cases it can be challenging to determine whether overgrowth is confined to the vascular component or also involves non-vascular tissues [5]. This uncertainty may result from a global increase in regional volume, secondary tissue deformation or growth disturbances of structures next to the vascular malformation, and segmental increase in perfusion. Moreover, there may be intrinsic limitations of imaging techniques in reliably distinguishing the vascular from the surrounding non-vascular tissue proliferation. This clinical overlap of such conditions makes molecular testing imperative, given that some of the disease entities may have a targeted biological therapy (e.g., *PIK3CA* inhibitors in PROS) [8].

In this study, we describe a cohort of children and adolescents with non-*PIK3CA* VMs associated with clinical overgrowth, highlighting their clinical features and exploring genotype-phenotype correlations.

## Methods

### Selection of participants

This study was designed as a multicentric retrospective analysis. Clinical and genetic data were collected from patients evaluated at the Pediatric Clinical Genetics Unit of the Regina Margherita Children’s Hospital (Torino), Fondazione Policlinico Universitario Agostino Gemelli (Roma), Santa Maria della Misericordia Hospital (Udine), San Bortolo Hospital (Vicenza), and the Bambino Gesù Children’s Hospital (Roma). Genetic data were analyzed at the Medical Genetics Unit of Policlinico Hospital (Bari). We selected patients according to established inclusion criteria: (1) diagnosed with VMs; (2) overgrowth of a body region; (3) harboring pathogenic or likely pathogenic variants in genes of the PI3K/AKT/mTOR signalling pathway, excluding *PIK3CA*, or other pathways involved in vascular proliferation. Available relatives carrying the same variant were also included. Patients without an identifiable pathogenic variant were excluded, as well as patients with isolated VMs (i.e., patients with isolated VMs and no evidence of body overgrowth beyond the malformation).

### Genetic analysis

Genetic testing was performed in all patients using peripheral blood (*n* = 12), buccal swab (*n* = 2), or biopsy samples from affected tissue (*n* = 27), according to clinical presentation and family history. For all participants, gDNA was extracted from peripheral venous blood, buccal swab, or from fresh or frozen biopsies of affected body regions using the QIAamp Mini Kit (Qiagen, Hilden, Germany), according to the manufacturer’s instructions. DNA quantity and quality were assessed using a BioSpectrometer Plus (Eppendorf, Hamburg, Germany).

DNA samples were analysed by Next Generation Sequencing (NGS) using two AmpliSeq Illumina Custom DNA Panels (Illumina, 2021), including 17 genes in the PI3K/AKT/mTOR pathway (*RASA1, PIK3CA, PIK3R1, PIK3R2, TEK, TSC2, GNAQ, TSC1, MTOR, PTEN, AKT3, AKT2, DEPDC5, AKT1, CCND2, NPRL3, GNA11*). The reading depth was > 2000 reads per base. Generally, NGS platforms using targeted exomic multi-gene panels were capable of detecting variants with a limit of detection as low as 5%, and this sensitivity could be further enhanced to even lower levels with a minimum read coverage of 1000×. Sequencing runs and variant interpretation were performed as previously described [6]. Germline variants and somatic variants with VAF > 15% identified by NGS were subsequently verified and confirmed by Sanger sequencing.

Bioinformatic analysis employed BWA-MEM and GATK (commercial pipelines) for secondary data processing. Variant filtering, prioritization, and annotation of single nucleotide variants (SNVs) and small insertions/deletions (indels) were conducted using Alamut Visual Plus 1.13, Emedgene v.36.10.0, Illumina Connected Analytics 2.43.0, and Illumina Connected Insights 5.3.1. Variants were annotated following the HGVS nomenclature and classified according to ACMG guidelines, integrated with current evidence from the scientific literature. The reference genome used was hg19, and variant databases included dbSNP 138 and HGMD (Human Gene Mutation Database).

### Clinical data collection

Clinical data were prospectively collected and recorded in a dedicated database structured into six predefined domains: (1) demographic and baseline characteristics; (2) prenatal features; (3) perinatal data; (4) clinical phenotype; (5) variants found and tissue analysed; (6) treatments and clinical management. Overgrowth was assessed by standardized clinical examination, including anthropometric measurements, and further characterized by imaging studies when available to define extent and tissue-specific involvement. Predefined variables included involvement of adipose, muscular, and skeletal tissues, as well as internal organs or central nervous system involvement and epilepsy.

VMs were classified according to the 2018 updated International Society for the Study of Vascular Anomalies (ISSVA) classification [9]. To ensure a consistent phenotypic definition across the cohort, only patients presenting with VM associated with overgrowth were included. Overgrowth was defined as a clinically appreciable increase in the size of a body region compared to the contralateral side or to expected age-related standards, involving one or more tissue components beyond the vascular lesion itself. The assessment of regional overgrowth was based on a combination of clinical and instrumental evaluations. Clinical examination included the assessment of asymmetry in limb girt or length or overall lateralized regional volume disproportion for symmetric parts of the body. When clinically indicated, imaging studies were performed to further characterize tissue involvement and asymmetry, including ultrasound, doppler ultrasound, magnetic resonance imaging (MRI), computed tomography (CT), and conventional radiography. These investigations aimed to evaluate differences in adipose, muscular, skeletal, and other tissue components between affected and unaffected or contralateral regions. Given the intrinsic difficulty in distinguishing true tissue overgrowth from “apparent” enlargement (e.g., secondary to vascular expansion or hemodynamic factors), both true and apparent overgrowth were considered for inclusion when supported by clinical and/or imaging findings. It is acknowledged that this inclusive definition encompasses both true mesenchymal overgrowth and regional enlargement attributable to vascular expansion or secondary tissue effects. In patients with *PTEN* variants, macrocrania was considered as a regional overgrowth phenotype in the context of the broader *PTEN* hamartoma tumor syndrome spectrum; similarly, hemimegalencephaly in *GNA11* and *AKT3* patients was classified as regional overgrowth due to cortical dysplasia associated with somatic mosaicism, rather than vascular-driven tissue expansion.

Each case was tentatively assigned a syndromic diagnosis based on clinical phenotype alone, independently of molecular findings, when possible. Symptoms and complications were systematically recorded, including involvement of adipose, muscular (compression, fatigue), and skeletal tissues (erosions, focal bone lesions), as well as internal organ manifestations (organ failure, central nervous system involvement with associated epilepsy). Functional outcomes were assessed based on pain, limitations in activities of daily living, and mobility impairment. Hemostatic parameters, including pro-thrombin and thrombin time, d-dimer, fibrinogen, and international normalized ratio (INR), were evaluated at least once in all patients and interpreted in relation to clinical history and the characteristics of the vascular malformation.

## Results

This retrospective study identified 41 patients (20 males and 21 females) with non-*PIK3CA* VMs associated with a clinical spectrum of tissue overgrowth. The mean age at first clinical assessment was 9.0 ± 2.3 years, while the mean age at diagnosis was 12.1 ± 1.4 years.

### Genotype

The variants identified were in *TEK* (24.4%, 10 patients), *GNAQ* and *GNA11* (21.9%, 9 patients each), *PTEN* (14.6%, 6 patients), *RASA1* (12.2%, 5 patients), *AKT3* and *EPHB4* (2.4%, 1 patient each). Of these variants, 34.1% were germline (14/41) and 65.9% (27/41) were somatic. There were 12 of the 14 germline cases had relatives tested: six of them (15.4%) had the inherited pathogenic variant (one case each involving *TEK*, *EPHB4,* and *RASA1*, and three *PTEN*). All germline variants were identified on peripheral blood samples, except for two cases in which the variant was first detected on DNA from buccal swab (given the buccal/cranial involvement of VM or overgrowth) and subsequently confirmed on blood-extracted DNA. Somatic variants were identified on affected-tissue biopsies and then verified on blood-extracted DNA: none of such cases showed a somatic variant later reclassified as germline nor detected in blood DNA. Table 1 summarised the genetic findings for all patients.

**Table 1:** Molecular characteristics of affected individuals

ID	Sex	Gene	Variant	Aminoacid change	Variant origin	Variant information	Sample
1	F	*AKT3*	c.241_243dup	p.Thr81dup	Mosaic	8.0%	Skin biopsy
2	M	*EPHB4*	c.1054C>T	p.Arg352*	Germline	Maternal	Blood
3	F	*GNA11*	c.547C>T	p.Arg183Cys	Mosaic	4.4%	Skin biopsy
4	M	*GNA11*	c.547C>T	p.Arg183Cys	Mosaic	9.13%	Skin biopsy
5	F	*GNA11*	c.547C>T	p.Arg183Cys	Mosaic	7.0%	Skin biopsy
6	M	*GNA11*	c.547C>T	p.Arg183Cys	Mosaic	3.0%	Skin biopsy
7	F	*GNA11*	c.547C>T	p.Arg183Cys	Mosaic	2.8%	Skin biopsy
8	F	*GNA11*	c.547C>T	p.Arg183Cys	Mosaic	19.0%	Skin biopsy
9	M	*GNA11*	c.547C>T	p.Arg183Cys	Mosaic	6.0%	Skin biopsy
10	F	*GNA11*	c.547C>T	p.Arg183Cys	Mosaic	3.0%	Skin biopsy
11	F	*GNA11*	c.547C>T	p.Arg183Cys	Mosaic	2.2%	Skin biopsy
12	M	*GNAQ*	c.548G>A	p.Arg183Gln	Mosaic	9.0%	Skin biopsy
13	F	*GNAQ*	c.548G>A	p.Arg183Gln	Mosaic	6.0%	Skin biopsy
14	F	*GNAQ*	c.548G>A	p.Arg183Gln	Mosaic	3.0%	Skin biopsy
15	M	*GNAQ*	c.548G>A	p.Arg183Gln	Mosaic	3.0%	Skin biopsy
16	F	*GNAQ*	c.548G>A	p.Arg183Gln	Mosaic	3.0%	Skin biopsy
17	F	*GNAQ*	c.548G>A	p.Arg183Gln	Mosaic	4.0%	Skin biopsy
18	F	*GNAQ*	c.548G>A	p.Arg183Gln	Mosaic	4.0%	Skin biopsy
19	M	*GNAQ*	c.548G>A	p.Arg183Gln	Mosaic	2.7%	Skin biopsy
20	F	*GNAQ*	c.548G>A	p.Arg183Gln	Mosaic	2.2%	Skin biopsy
21	M	*PTEN*	c.546_549delAAAG	p.Lys183Ilefs*15	Germline	Paternal	Blood
22	M	*PTEN*	c.697C>T	p.Arg233Ter	Germline	De novo	Blood
23	M	*PTEN*	c.545dup	p.Leu182Phefs*8	Germline	De novo	Blood
24*	F	*PTEN*	c.491del	p.Lys164ArgfsTer3	Germline	Paternal	Blood
25*	M	*PTEN*	c.491del	p.Lys164ArgfsTer3	Germline	Paternal	Blood
26*	M	*PTEN*	c.491del	p.Lys164ArgfsTer3	Germline	Unknown	Blood
27	M	*RASA1*	c.543G>A	p.Trp181Ter	Germline	De novo	Buccal swab
28	M	*RASA1*	c.1980_1981dup	p.Lys661Ilefs*18	Germline	De novo	Blood
29*	M	*RASA1*	c.768C>A	p.Tyr256Ter	Germline	Maternal	Blood
30*	M	*RASA1*	c.768C>A	p.Tyr256Ter	Germline	Unknown	Blood
31	F	*RASA1*	c.1253+1G>A	N/A	Germline	De novo	Blood
32	F	*TEK*	c.2545C>T	p.Arg849Trp	Germline	Paternal	Blood
33	M	*TEK*	c.2744G>T	p.Arg915Leu	Germline	De novo	Blood
34	M	*TEK*	c.2740C>T	p.Leu914Phe	Mosaic	5.2%	Skin biopsy
35	F	*TEK*	c.2740C>T	p.Leu914Phe	Mosaic	7.0%	Skin biopsy
36	F	*TEK*	c.2740C>T	p.Leu914Phe	Mosaic	8.0%	Skin biopsy
37	F	*TEK*	c.2740C>T	p.Leu914Phe	Mosaic	8.0%	Skin biopsy
38	M	*TEK*	c.3308_3314dup	p.Thr1106Glufs*6	Mosaic	3.0%	Skin biopsy
39	F	*TEK*	c.3339 T>A	p.Tyr1113*	Mosaic	8.0%	Skin biopsy
40	F	*TEK*	c.2740C>T	p.Leu914Phe	Mosaic	11.0%	Buccal swab
41	M	*TEK*	c.2740C>T	p.Leu914Phe	Mosaic	5.0%	Skin biopsy

*Patient #26 is the affected father of patients #24 and#25; patient #30 is the affected mother of patient #29

*F* female, *M* male

### Phenotype

The phenotypes observed in our cohort were highly heterogeneous, ranging from isolated vascular malformations with localized overgrowth confined to a single anatomical district to complex, multisystem presentations involving multiple body regions and relevant multiorgan complications. Given the heterogeneity of clinical presentation, each patient was characterized based on the tissue involved in overgrowth based on clinical evaluation and imaging studies. Anomalies in skeletal growth were documented in 19 patients (46.3%), predominantly in association with variants in *GNAQ* and *GNA11*, but also reported in individuals with *RASA1*, *TEK*, and *PTEN* mutations. Leg length discrepancies were observed in 17 cases (15 with overgrowth and two with growth restriction of the affected limb). One patient (#41) exhibited an atypical presentation in which the marked serpiginous vascular overgrowth, characterised by prominent coil-like vascular structures, resulted in impaired development of the affected limb with relevant limb length discrepancy and muscular hypotrophy. A similar, but clinically milder pattern of segmental undergrowth was observed in patient #9, carrying a *GNA11* variant, presented with cutis marmorata telangiectatisa congenita associated.

Apparent muscular or soft tissues overgrowth was observed in 25 cases (60.9%), mostly associated with *GNAQ* and *GNA11* variants. Adipose overgrowth was identified in 4 individuals (10.7%) and was associated with variants in *PTEN*, *RASA1* and *GNAQ*. Macrocrania was present in eight patients (19.5%), mostly associated with *PTEN* variants (five cases). Overgrowth of internal organs was observed in only three individuals (7.3%). In a patient (#33) with a germline variant in *TEK*, visceral overgrowth involved the colon and appeared to be related to extensive vascular malformations affecting the same organ. The other two patients, with variants in *GNA11* (#5) and *AKT3* (#1), both exhibited hemi-megalencephaly.

Among the 41 patients in the cohort, four individuals exhibited clinically relevant haemostatic or thrombotic complications. Patient #6 (with a variant in *GNA11*) developed cerebral ischemia with subsequent epilepsy and right hemiparesis at five years of age. Patients #36 and #37 (with variants in *TEK*) reported recurrent pain episodes associated with intralesional thrombosis and phlebolithiasis formation, while patient #38 (with a variant in *TEK*) experienced episodes of acute intralesional thrombosis. No other patients showed thrombotic events or coagulation abnormalities at blood tests. Regarding symptom burden, 18 patients (43.9%) reported some functional limitation, ranging from fatigue and difficulties for high-demand physical activities to gait impairment, transient pain episodes, and visual deficits. Psychosocial impact was formally reported by only one patient (#40), however, this likely underestimated the true burden, as psychological distress related to disfiguring lesions was often underreported to clinicians and remained insufficiently addressed in routine clinical assessment.

**Fig. 1:**
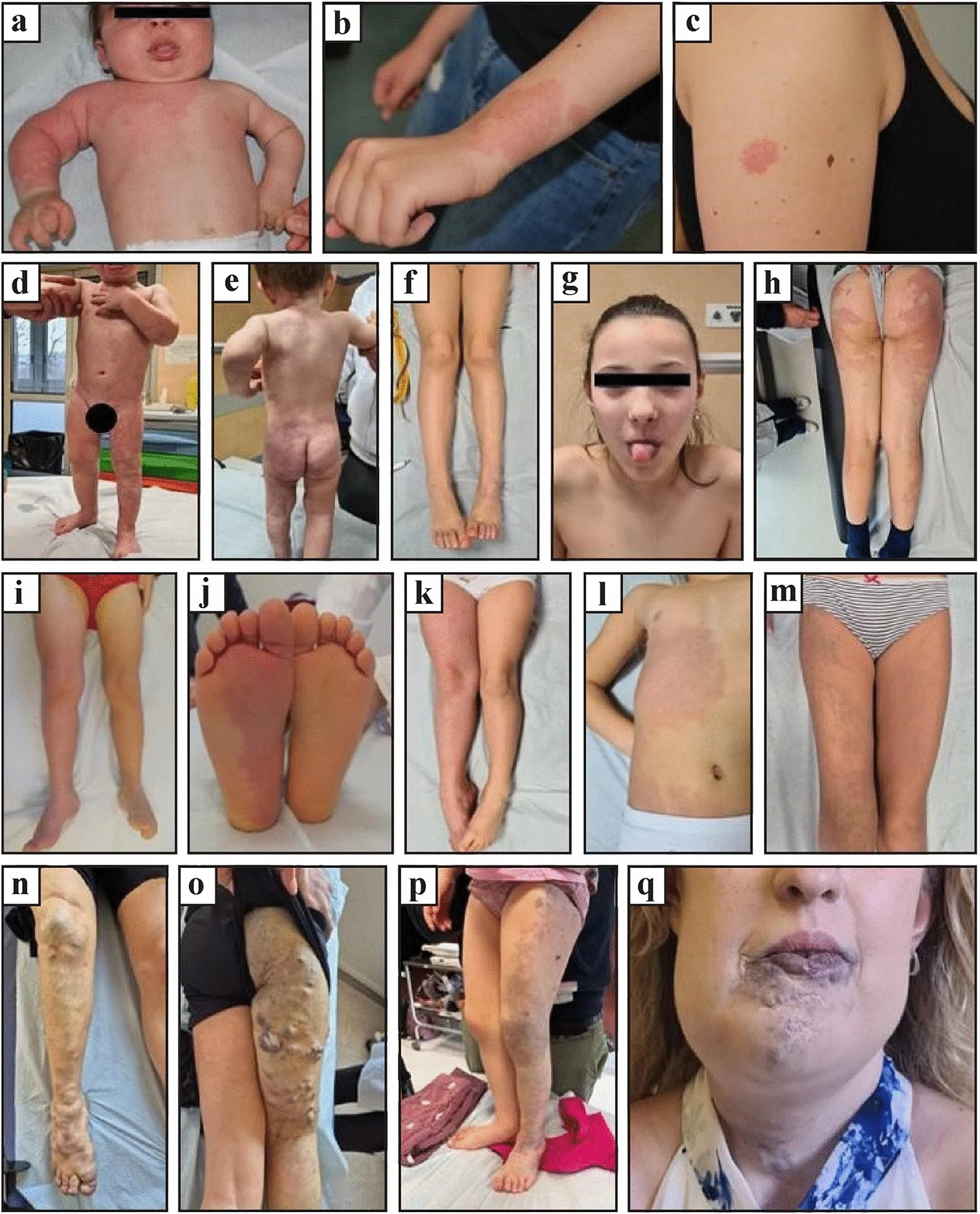
Clinical cases with various vascular malformations caused by different genes. **a** Patient #29 with *RASA1* variant and Parkes-Weber syndrome; **b–c** Patient #30, mother of patient #29, with flat capillary malformation on the left wrist, right shoulder and left thigh; **d–e** Patient #9 with *GNA11* variant, cutis marmorata telangectasica and mild hypoplasia of the affected left lower limb; **f–g** Patient #11 with *GNA11* variant, capillary malformation distributed across the left side of the body and face and left hemihypertrophy; **h** Patient #1 with *AKT3* variant, hemimegalencephaly and capillary malformation on the lateral and posterior surfaces of both lower limbs, predominantly on the right; **i–j** Patient #19 with *GNAQ* variant, capillary malformation on the entire right lower limb, right lower limb hyperplasia (< 1 cm); **k–m** Patient #16 with *GNAQ* variant, lateralized overgrowth, capillary malformation of the right lower limb, the right hemiabdomen and hemithorax, ectasia of the saphenous axis of the right lower limb; **n–o** Patient #41 with *TEK* variant, venous malformation of the right lower limb with serpiginous vascular overgrowth and hypoplasia of the right lower limb with muscle atrophy; **p** Patient #37 with *TEK* variant, venous malformation of the left lower limb, numerous serpentine vascular structures extending along the entire left lower limb within the subcutaneous soft tissue; **q** Patient #40 with *TEK* variant, venous malformation of the right side of the face, left eyelid ptosis and soft tissue hypertrophy of the palate, tongue, and left cheek

Table 2 summarised the key phenotypic features and symptoms of all patients. Figure 1 showed clinical presentations of various individuals. A “syndromic” diagnosis was established in 26 patients (63.4%), while in the remaining cases, a precise syndromic classification could not be assigned, as no currently defined syndrome fully matched the observed clinical presentation, despite complex, multisystemic phenotypes rather than isolated manifestations, thus fulfilling the broader conceptual definition of a syndrome as a recurrent constellation of co-occurring clinical signs and symptoms. Considering the distribution of clinical diagnoses across genes, *GNA11* variants were found in patients classified as having Klippel-Trenaunay syndrome, diffuse capillary malformation with overgrowth (DCMO), Sturge-Weber syndrome, phakomatosis pigmentokeratotica, and in several cases without a defined syndromic classification. Variants in *GNAQ* were identified in patients with DCMO, Klippel-Trenaunay syndrome, Sturge-Weber syndrome, phakomatosis pigmentokeratotica, and unclassified phenotypes. In patients with *GNA11* and *GNAQ* variants assigned a diagnosis of Klippel-Trenaunay syndrome, the diagnosis was established based on the coexistence of a capillary malformation (port-wine stain-like, segmental distribution), limb hypertrophy, and at least one additional vascular anomaly (lymphatic or venous malformation) in accordance with ISSVA criteria. Patients classified as DCMO presented with diffuse, non-dermatomal capillary malformations associated with lateralized or segmental overgrowth, without venous or lymphatic components. *TEK* variants were observed in patients with Klippel-Trenaunay syndrome, blue rubber bleb nevus syndrome, and in cases without a defined syndromic classification. *RASA1* variants were identified in patients with Parkes-Weber syndrome, Klippel-Trenaunay syndrome, and unclassified phenotypes. *PTEN* variants were observed in patients with Bannayan-Riley-Ruvalcaba syndrome, Cowden syndrome, and in one case without a defined syndromic classification.

**Table 2:** Main clinical characteristics and syndromic classification of the affected individuals

ID	Gene	Syndromic classification	OMIM classification	Body region	ISSVA classification [9]	Clinical characterization	Overgrowth	Other features	Symptoms and complications
1	*AKT3*	–	–	Head, lower limbs	CM	Diffuse flat angioma on the lateral and posterior surfaces of both lower limbs	Hemi-megalencephaly	–	Seizures
2	*EPHB4*	CM-AVM2	#618,196	Head, trunk, left upper limb, right lower limb	CM	CM of the scapula and left upper limb. Small subcentimeter capillary malformations on the hands, face, lips, thigh, and right foot. Nasal mucosal ectasia	–	–	Recurrent nosebleeds
3	*GNA11*	–	–	Whole body	CM	Diffuse CM (patchy)	Mild hypoplasia of the right lower limb	Bilateral conjunctival melanosis; limb length discrepancy	Not reported
4	*GNA11*	KTS	#149,000	Trunk, limbs	CM, VM, AM	CM of trunk, right lower and upper limbs. Ectasia and tortuosity of the veins of the superficial area of the right lower limb (without embryonic veins). Reduction in calibre of the arterial tree of the right lower limb	–	–	Easy fatigue; limitation in high-demand physical activities
5	*GNA11*	DCMO	–	Whole body	CM	CM on the right side of the body, alternating with predominantly pink angiomatous areas, distributed throughout the body	Left lateralized overgrowth; mild left hemi-megalencephaly	Limb length discrepancy	Not reported
6	*GNA11*	KTS	#149,000	Whole body	CM	Diffuse capillary malformation of the face, trunk, and predominantly upper limbs	Lateralized overgrowth	Exophthalmos of the right eye; cerebral malformation with some hypotrophic perieto-occipital gyri; blue nevus; limb length discrepancy	Cerebral ischemia at 5 y of age with subsequent epilepsy and hemiparesis; intellectual disability; motor impairment
7	*GNA11*	SWS	#185,300	Right hemibody	CM	Diffuse CM on the right side of the body (more subtle on the lower limb) with involvement of the first two trigeminal branches of the right side of the face, associated with an anemic area adjacent to the capillary malformation of the right arm	–	Right hemispheric pial angiomatosis	Not reported
8	*GNA11*	DCMO	–	Whole body	CM	Diffuse superficial CM affecting the face, part of the trunk, and parts of the lower and upper limbs	Right foot enlarged in length and circumference	Left eye glaucoma operated on at 4 mon of age; limb length discrepancy; nasal pyogenic granuloma	Visual impairment
9	*GNA11*	–	–	Trunk, left lower limb	CM	CM (marbled erythematous-violet macules) spread across the upper, scapular, pectoral, and left lower limb	Mild hypoplasia of the left lower limb	Limb length discrepancy	Gait abnormalities
10	*GNA11*	PPK	–	Right limbs	CM	Metameric CM of the right upper and lower limbs	–	Oculodermal melanocytosis; autoimmune thyroiditis; hypoadrenocortical dysplasia; right-sided convex scoliosis of the lumbar spine; obesity	Neurodevelopmental delay; seizures; chronic cervical pain
11	*GNA11*	DCMO	–	Left hemibody	CM	CM on the left side of the body and face	Left lateralized overgrowth	Limb length discrepancy	Limitation in high-demand physical activities
12	*GNAQ*	DCMO	–	Whole body	CM, VM	Diffuse capillary malformation (limbs, trunk, head). Venous ectasia of the lower limbs	Mild localized overgrowth of areas with vascular malformation	–	Limitation in high-demand physical activities
13	*GNAQ*	SWS	#185,300	Head, lower limbs	CM	Facial cutaneous-mucosal capillary malformations. Episcleral telangiectasias	Macrocephaly; hemifacial overgrowth; lower limb lateralized overgrowth	Brain malformations; limb length discrepancy	Seizures
14	*GNAQ*	–	–	Right upper limb, trunk	VM	VM on right upper limb, right scapula and shoulder, right pectoralis major	–	–	Not reported
15	*GNAQ*	DCMO	–	Right lower limb	CM	Planar angioma of the right lower limb	Right lower limb hyperplasia	Limb length discrepancy	Motor dyspraxia with difficulty with motor coordination and balance
16	*GNAQ*	KTS	#149,000	Right hemibody	CM, VM	Angioma of the right lower limb. Ectasia of the saphenous axis of the right lower limb. Angiomatous lesion distributed up to the midline of the abdomen and thorax	Right lateralized overgrowth	Limb length discrepancy	Gait abnormalities
17	*GNAQ*	DCMO	–	Head, right limbs	CM	CM of the right cheek region with a medial border to the philtrum, of a large portion of the scalp, of the left temporal region, right upper limb, right hemodorsum, right trochanteric region, and flexor surface of the right leg	Right upper and lower limb lateralized overgrowth; borderline high head circumference	Limb length discrepancy	Gait abnormalities
18	*GNAQ*	PPK	–	Whole body	CM	Diffuse capillary malformation on the right side of the face, all four limbs, trunk, upper and lower limbs	Overgrowth of the cheeks and lips; macrocephaly	Dermal melanosis predominantly on the left flank, shoulders, and thighs; involvement of the bilateral ocular conjunctiva; glaucoma of the right eye; pial and infratentorial angiomatosis	Mild neurodevelopmental delay; visual impairment
19	*GNAQ*	DCMO	–	Right lower limb	CM	CM on the right lower limb	Right lower limb lateralized overgrowth	Limb length discrepancy	Gait abnormalities
20	*GNAQ*	DCMO	–	Head, lower limbs	CM	Diffuse patchy CM of the lower limbs, with some patches on the back of neck and face	Right lower limb lateralized overgrowth	Limb length discrepancy	Not reported
21	*PTEN*	BRRS	#158,350	Whole body	VM, LM	Microcystic LM of the first toe and right calf. Venous/lymphatic vascular malformations at the base of the proximal phalanx of the third finger of the right hand	Multiple lipomas	Multiple benign thyroid nodules; sessile gastric micropolyps	Easy fatigue; limitation in daily activities
22	*PTEN*	CS	#158,350	Head, abdomen, right hand	VM	VM of the left inguinal region and right hand. Left frontal cavernoma	Macrocephaly	Cortical dysplasia with polymicrogyria; thyroid nodules	Seizures; neurodevelopmental delay; hyperactivity
23	*PTEN*	–	–	Head, abdomen, right lower limb	VM, AM	Mixed VM of the right lower limb	Macrocephaly; umbilical lipoma	–	Not reported
24	*PTEN*	BRRS	#158,350	Head, lower limbs	AVM, LM	AVM of the left lower limb. Microcystic LM of the right lower limb	Macrosomia at birth; macrocephaly	Chiari malformation type 1	Episodic pain on the lower limbs
25	*PTEN*	BRRS	#158,350	Head, right limbs	VM	VM of the right forearm. Linear VM of the right foot. Cavernous angioma in the frontal region	Macrosomia at birth; macrocephaly	Multiple thyroid micronodules	Seizures
26	*PTEN*	CS	#158,350	Head	VM	Ectatic VM of the tongue	Macrocephaly	–	Not reported
27	*RASA1*	–	–	Upper right limb, thorax	VM	Angiomatosis of the right hemithorax and right upper limb	Soft tissue overgrowth of the right upper limb with lateralized overgrowth	–	Not reported
28	*RASA1*	–	–	Left lower limb	VM	Multiple cutaneous angiomas	Left lower limb lateralized overgrowth	Pulmonary valvular stenosis; limb length discrepancy	Gait abnormalities
29	*RASA1*	PWS	–	Whole body	CM, AVM	Diffuse capillary malformations. Arteriovenous malformation of the right lower limb	Right lower limb lateralized overgrowth	Cardiomegaly; neonatal chylothorax; limb length discrepancy	Neurodevelopmental delay; easy fatigue; limitation in daily activities; seizures
30	*RASA1*	–	–	Left limbs	CM	CM on the left wrist, right shoulder and left thigh	–	–	Not reported
31	*RASA1*	KTS	#149,000	Abdomen	CM, VAM	CM in the pubic area. Multiple diffuse ectatic angiomas	Fibro-lipomatous overgrowth in the pubic area	Two angiokeratomas	Not reported
32	*TEK*	–	–	Whole body	VM, LM	Diffuse venous malformations. Diffuse multi-district lymphangiomas	Paravertebral schwannoma	–	Easy fatigue; limitation in daily activities
33	*TEK*	BRBNS	#112,200	Whole body	VM	VM of the limbs and the colon	–	Hyperlaxity. kyphoscoliosis	Easy fatigue; limitation in high-demand physical activities
34	*TEK*	–	–	Foot	Vascular tumour	KHE of the foot	Undergrowth (thinning of the muscles of the foot)	–	Gait-associated pain; limitation in high-demand physical activities
35	*TEK*	–	–	Hand	VM	Multiple VM on the right hand	Lateralized overgrowth of the hand	–	Limitation in daily activities
36	*TEK*	–	–	Abdomen, right lower limb	VM	VM with a diffuse superficial network of lesions extending from the right lower limb to the ipsilateral flank	Right foot overgrowth	–	Recurrent pain episodes; limitation in high-demand physical activities
37	*TEK*	KTS	#149,000	Left lower limb	VM	VM of the left lower limb, with numerous serpentine vascular structures extending along the entire left lower limb within the subcutaneous soft tissue	Left lower limb lateralized overgrowth	Limb length discrepancy	Recurrent pain episodes; easy fatigue; limitation in daily activities; gait abnormalities
38	*TEK*	–	–	Thorax	VM	Dorsal VM	Apparent overgrowth of the tissues surrounding AVM	Recurrent episodes of intralesional thrombosis with pain	Pain
39	*TEK*	–	–	Head	VM, CM	Mixed venous-capillar malformation on the left lower lip. Segmental cutaneous telangiectasia on the left mandibular arch	Soft tissue overgrowth on the left cheek	–	Not reported
40	*TEK*	–	–	Head	VM	VM of the right side of the face extending to the canthus	Soft tissue hypertrophy of the palate, tongue, and left cheek	Left eyelid ptosis	Persistent pain; feeding and chewing difficulties; social limitation due to disfiguring effects
41	*TEK*	KTS	#149,000	Limb	VM	VM of the right lower limb	Hypoplasia of the right lower limb with muscle atrophy, circumferential lateralized overgrowth (apparent) due to AVM	Limb length discrepancy	Persistent pain; gait abnormalities; limitation in daily activities

When available, OMIM classification is reported after the syndrome name

*Clinical syndromic classifications refer to the classification given on the sole phenotype before molecular tests results

*ISSVA* International Society for the Study of Vascular Anomalies, *VM* venous malformation, *AM* arterial malformation, *CM* capillary malformation, *AVM* arteriovenous malformation, *LM* lymphatic malformation, *OMIM* online mendelian inheritance in man, *KHE* Kaposiform hemangioendothelioma

Therapeutic management across the cohort was heterogeneous: six patients (14.6%) underwent combined therapeutic approaches, including four individuals with *TEK*-related disease: one (#32) treated with surgery plus sclerotherapy, two (#36, #38) with sclerotherapy combined with pharmacological therapy, and one (#40) with surgery followed by medical treatment. Combined strategies were also adopted in one patient with a *GNAQ* variant (#12) and one with a *GNA11* variant (#6), both of whom received laser therapy in association with surgery. Surgical intervention alone was performed in nine patients (21.9%), including four patients with variants in *TEK*, two in *PTEN*, one in *GNA11*, and two in *GNAQ*. Importantly, in all, but one case, surgery was not resolutive, and patients experienced subsequent relapse of the overgrowth in the treated area. Sclerotherapy was administered to seven patients, mostly with variants in *TEK* variants (four cases) or *PTEN* (three cases). Laser therapy represented the primary intervention in five cases (11.1%), three with variants in *GNAQ* and two with variants in *GNA11*.

Six patients (14.6%), all with variants in *TEK*, received systemic pharmacological treatment. Propranolol was employed in one case, achieving a favourable clinical response with symptomatic improvement and reduction in lesional volume. Sirolimus was administered in four patients, but discontinued in all of them due to severe recurrent infections in one case, or because of absent or limited clinical benefit (three cases). Three of the patients treated with sirolimus were subsequently transitioned to a selective *PIK3CA* inhibitor (Alpelisib), which led to clinical and volumetric improvement in two, while one required treatment cessation due to a cutaneous retarded hypersensitivity adverse reaction. One patient with a variant in *TEK* was treated exclusively with Alpelisib, showing satisfactory control of symptoms with no adverse effects. Details of medical therapy are provided in Table 3.

**Table 3:** Medical therapy and its effect in treated patients

ID	Variant	Previous treatments	Therapy	Age at therapy onset	Duration of therapy	Efficacy	Side effects
35	*TEK*: c.2740C>T	Sclerotherapy at 8 y	Alpelisib	10 y	2 y (ongoing)	Symptoms remission, volume stabilization; dimensional stability of the lesion	None
36	*TEK*: c.2740C>T	Sclerotherapy at 6 y	Sirolimus	7 y	3 y	Slight increase of the lesion; persistence of pain episodes	Neutropenia with minor recurrent infections
			Alpelisib	11 y	1 y (ongoing)	Reduction of pain episodes	Not reported
37	*TEK*: c.2740C>T	–	Sirolimus	3 y	6 mon	Progression of the lesion	Recurrent infections
			Alpelisib	4 y	6 mon (ongoing)	Reduction of symptoms	None
38	*TEK*: c.3308_3314dup	–	Sirolimus	3 y	1 y	Progression of the lesion during treatment	None
			Alpelisib	4 y	1 y (ongoing)	Reduction of the frequency of pain episodes; slight dimension progression of the lesion	None
39	*TEK*: c.3339 T>A	–	Propanolol	5 mon	2 y	Reduction of the volume, remission of pain	None
40	*TEK*: c.2740C>T	Multiple sclerotherapy session since childhood	Sirolimus	17 y	3 y	Progression of the lesion during treatment	Recurrent severe infections*
			Alpelisib	20 y	1 y	Dimensional stability of the malformation	Hypersensitivity*

*Adverse reactions effects requiring therapy discontinuation

## Discussion

Available data on vascular malformations with overgrowth have largely focused on gene-specific descriptions, often considering individual conditions in isolation. In contrast, our study adopts a paediatric and clinical oriented perspective and demonstrates that non-*PIK3CA* vascular malformations with overgrowth represent a highly heterogeneous group with substantial phenotypic overlap across different genetic etiologies. While PROS are well-defined and relatively prevalent, non-*PIK3CA* conditions remain less systematically characterized and are frequently underrecognized. Our findings highlight that these disorders consistently share a huge overlap in clinical features in real-world practice, reinforcing the need for broad molecular testing strategies and comprehensive gene panels to achieve accurate diagnosis [6].

A key finding emerging from our cohort is the limited reliability of phenotype-based syndromic classification. Although a clinical syndromic diagnosis could be assigned in 63.4% of patients based on phenotype only, a substantial proportion remains difficult to be classified, and, importantly, the same clinical entities were associated with multiple genes, while the same gene gave rise to different syndromic diagnoses. Variants in *GNAQ* and *GNA11*, for example, were identified across a wide spectrum including DCMO, Klippel-Trenaunay syndrome, Sturge-Weber syndrome, and phakomatosis pigmentokeratotica, as well as in non-classifiable phenotypes. Similarly, *TEK* and *RASA1* variants spanned both well-defined syndromes and unclassified presentations. These observations underscore the intrinsic limitations of a phenotype-first approach and support a shift toward molecularly driven classification frameworks in vascular malformations.

A first level of complexity in interpreting these disorders arises from the coexistence of mosaic and germline variants, which accounted, respectively, for 65.9% and 34.1% of cases in our cohort. Notably, we observed a discordance between clinical suspicion and molecular findings in several cases. Segmental distribution and apparently sporadic presentation often suggested mosaicism, prompting tissue biopsy; however, germline variants were subsequently identified. Conversely, familial cases revealed marked intrafamilial variability, with mildly affected or apparently asymptomatic relatives carrying the same pathogenic variant. These findings emphasize that clinical pattern alone is insufficient to infer mutational origin and parental testing should be considered, particularly when the VAF in the proband and the clinical presentation are consistent with possible germline or low-level germline mosaicism [10]. However, in the absence of parental symptoms or clinical findings, this decision may reasonably be deferred pending complete molecular characterization of the proband. Mosaicism itself represents a major driver of phenotypic heterogeneity [11]. Postzygotic mutations occurring at different developmental stages result in variable tissue distribution and clinical expression [12]. In this context, our data further demonstrate that VAF is not predictive of disease severity: low-level mosaic variants may be associated with severe phenotypes, while higher VAFs may correspond to milder presentations. This finding reinforces the concept that disease expression reflects not only the proportion of mutated cells, but also their spatial distribution and the specific tissues involved. From a diagnostic perspective, these observations confirm that sequencing of affected tissue significantly increases detection sensitivity, whereas blood-based testing alone may fail to identify low-level mosaicism [13]. Recurrent hotspot mutations were observed in *GNAQ* and *GNA11*, whereas *PTEN* and *RASA1* variants were more heterogeneous, in line with previous reports [14–16].

Beyond genetic architecture, our cohort highlights the complexity of genotype–phenotype correlations in non-*PIK3CA* vascular malformations. Although certain trends can be recognized, such as the predominance of capillary malformation phenotypes in *GNAQ*/*GNA11*-related conditions [14, 15], or the association of TEK variants with venous and combined malformations [17, 18], these patterns are not exclusive. Clinically, more severe complications appeared more frequently in patients carrying *TEK* and *GNA11* variants, although considerable variability persisted even within the same gene, further supporting the need for individualized assessment.

An important and underappreciated aspect emerging from our data concerns the interpretation of “overgrowth”. In clinical practice, regional enlargement is often attributed to true tissue overgrowth; however, our findings suggest that this distinction is not always straightforward. In several cases, apparent overgrowth likely reflected secondary effects of vascular malformations, including venous ectasia, increased perfusion, or structural deformation of adjacent tissues. Notably, macrocrania as observed in *PTEN*-related conditions likely reflects hamartomatous dysplasia rather than vascular-mediated tissue expansion, and hemimegalencephaly in *GNA11* and *AKT3* patients represents a cortical growth dysplasia rather than classical somatic overgrowth; these distinctions are clinically important and should be considered when comparing our cohort with series applying stricter definitions of overgrowth. This concept challenges the traditional view of overgrowth as a primary process and suggests that both true and apparent enlargement coexist within the same clinical spectrum. The difficulty in distinguishing these mechanisms also has implications for both diagnosis and therapeutic decision-making.

Consistent with this perspective, our cohort also included patients presenting with segmental undergrowth, always corresponding to the anatomical distribution of the vascular malformation. Although overgrowth is typically emphasized in these disorders, this finding supports the emerging concept that vascular malformations may also impair normal tissue development [19–23]. Hemodynamic disturbances, such as venous hypertension, microvascular shunting, or chronic hypoperfusion, may interfere with normal growth processes, leading to hypoplasia rather than hypertrophy. Our data extend previous observations to non-*PIK3CA* conditions, reinforcing the idea that vascular dysfunction can result in both overgrowth and undergrowth depending on timing, location, and severity [21].

From a clinical standpoint, the burden of disease in our cohort was substantial and not strictly correlated with lesion size or anatomical extent. Nearly half of patients reported functional limitations, including fatigue, pain, gait abnormalities, and visual impairment, while psychosocial impact was likely underestimated due to the lack of standardized assessment tools. These findings highlight the importance of comprehensive, multidisciplinary evaluation that includes not only anatomical characterization, but also functional and psychosocial dimensions [24, 25].

Therapeutic management was highly heterogeneous and reflected the absence of standardized treatment algorithms for these conditions. Surgical approaches were frequently non-curative, with most patients experiencing recurrence or progression after intervention. Similarly, sirolimus showed limited or variable efficacy and was often discontinued due to adverse effects or lack of clinical benefit. In contrast, treatment with the PI3K inhibitor alpelisib resulted in clinical improvement or disease stabilization in a subset of patients with *TEK* variants, despite the absence of *PIK3CA* mutations [26]. This observation suggests that different genetic defects may converge on shared downstream signalling pathways, providing a biological rationale for pathway-targeted therapies beyond classical PROS [26–29]. However, the lack of agents directly targeting upstream drivers, such as *TEK* or *GNAQ*/*GNA11,* highlights a significant unmet therapeutic need and an important area for future research. The favourable response to propranolol observed in one patient with a *TEK* variant is noteworthy, but should be interpreted with extreme caution. Current evidence supports the use of propranolol almost exclusively in infantile haemangiomas; its mechanism of action in venous malformations is not established, and this observation remains anecdotal.

From a diagnostic and organizational perspective, our findings support the use of broad NGS panels and emphasize the importance of tissue-based molecular analysis. The integration of clinical, radiological, and molecular data remains essential for accurate diagnosis and optimal management. Furthermore, the complexity of these conditions underscores the need for dedicated multidisciplinary teams and structured care pathways, including transition to adult services [30].

This study has several limitations. Its retrospective design limits the completeness and standardization of clinical data collection, and the robustness of statistical analysis. In addition, the inclusion of only genetically confirmed cases introduces a selection bias toward patients with identifiable pathogenic variants, potentially underrepresenting genetically unresolved conditions. Finally, the lack of standardized quality-of-life measures likely resulted in an underestimation of the true disease burden.

In conclusion, non-PIK3CA VMs with overgrowth represent a complex and heterogeneous group of disorders characterized by overlapping phenotypes, diverse genetic mechanisms, and variable clinical outcomes. Our findings demonstrate the limitations of phenotype-based classification, highlight the central role of molecular diagnosis, and support the concept of shared pathogenic pathways as a basis for emerging targeted therapies. Continued efforts to integrate clinical and molecular data will be essential to refine classification systems and improve patient care.

## Data Availability

The dataset supporting the conclusions of this article is included within the article. Additional information may be available upon reasonable request to the corresponding author.

## Declarations

### AI usage statement

No AI was used in any part of the research or in the writing of the article.
